# CAEP Position Statement – Management of devastating brain injuries in the emergency department: Enhancing neuroprognostication and maintaining the opportunity for organ and tissue donation

**DOI:** 10.1017/cem.2020.357

**Published:** 2020-09

**Authors:** Andrew Healey, Murdoch Leeies, Carmen Hrymak, Alecs Chochinov, Brian Grunau, Bojan Paunovic, Jeanne Teitelbaum, Lindsay C. Wilson, Sam D. Shemie

**Affiliations:** * Department of Medicine, Division of Emergency Medicine, McMaster University, Hamilton, ON; † Critical Care, William Osler Health System, Brampton, ON; ‡ Donation, Trillium Gift of Life Network, Toronto, ON; § Department of Emergency Medicine, Rady Faculty of Health Sciences, University of Manitoba, Winnipeg, MB; ¶ Organ Donation, Transplant Manitoba, Winnipeg, MB; ** Department of Emergency Medicine, University of British Columbia, Vancouver, BC; †† Centre for Health Evaluation and Outcome Sciences, Michael Smith Foundation for Health Research, Vancouver, BC; ‡‡ WRHA Critical Care, Critical Care Medicine, Winnipeg, MB; §§ Department of Internal Medicine, Rady Faculty of Health Sciences, University of Manitoba, Winnipeg, MB; ¶¶ Health Sciences Centre, Winnipeg, MB; *** Department of Neurology and Neurosurgery, Montreal Neurological Institute and Hospital, Montreal, QC; ††† Deceased Donation, Canadian Blood Services, Toronto, ON; ‡‡‡ Division of Critical Care, Montreal Children's Hospital, McGill University Health Centre, Montreal, QC; §§§ Deceased Donation, Canadian Blood Services, Montreal, QC

**Keywords:** Organ donation, brain injury, emergency medicine

## Abstract

The primary purpose of this statement is to improve neuroprognostication after devastating brain injury (DBI), with a secondary benefit of potential organ and tissue donation.

## EXECUTIVE SUMMARY

The primary purpose of this statement is to improve neuroprognostication after devastating brain injury (DBI), with a secondary benefit of potential organ and tissue donation.

## IN SCOPE

Adult patients with DBI who have had initiation of resuscitation measures, including intubation and mechanical ventilation.

## OUT OF SCOPE

Clinical decision-making regarding initiation of resuscitation measures after DBI.

## INTRODUCTION

*Devastating brain injury* is defined as a neurological injury (trauma, subarachnoid hemorrhage, stroke, hypoxic injury, etc.) that is assessed as an immediate threat to life or incompatible with good functional recovery and where early limitation or withdrawal of therapy is being considered.^1,2^ The outcomes for patients who present in the emergency department (ED) with DBI are often death or, in some cases, survival with extremely limited capacity. Consequently, many physicians consider admission to the intensive care unit (ICU) inappropriate as it is not only futile and challenging in the face of scarce ICU resources, but also a burden on patients and families by offering prolongation of what may be an inevitably poor outcome.

However, the management of DBI in the ED is evolving. Accurate prognostication in the early stages of DBI cases can be difficult, and rigorous, evidence-based prognostication strategies in patients with DBI are limited. Clinician variability in the withdrawal of life-sustaining treatments impacts patient outcomes. A recent multicentre retrospective cohort study of patients with severe DBI in Canadian level-one trauma centres identified significant variation in mortality across centres. Mortality rates were significantly impacted by varying approaches to withdrawal of life-sustaining measures (WLSM), highlighting deficiencies of early prognostic strategies in trauma.^3^ In-hospital mortality after intracerebral hemorrhage is significantly influenced by the variability in the rate at which treating hospitals use do-not-resuscitate orders, even after adjusting for case mix.^4^ To improve the quality of decision-making to better inform whether the patient can survive and recover, transfer to the ICU for a period of physiological support and observation is recommended.^1,2^

The optimal period for observation to establish greater confidence and accuracy in prognostication following DBI is not well established. The Neurocritical Care Society^1^ recommends a 72-hour observation period, during which physiological support can prevent unwarranted deterioration and allow sufficient opportunity for prognostic evaluation, care planning, and a more definitive determination of prognosis based on repeated examinations over time. The United Kingdom (Faculty of Intensive Care Medicine, Intensive Care Society, Neuroanaesthesia and Critical Care Society, Royal College of Emergency Medicine, Society of British Neurological Surgeons) recommends that the length of the observation period be based on a combination of clinical judgement, changes in neurological function, the degree of support required to maintain physiological stability, and communication with the patient's family to determine patient preferences.^2^

Though death may be the most likely outcome in many of these cases, a period of observation will further ensure the accuracy of this prognosis and avoid what may be an inappropriate limitation in care.^5^ The primary aim is to improve the ability to distinguish patients who may have the capacity to recover and survive. In addition, it allows for a timely referral to the provincial organ donation organization, ensuring that patients and their families are given sufficient opportunity to consider organ and tissue donation. A recent systematic review^6^ of ED deaths revealed that a substantial proportion (46.2–84%) of potential organ donors was missed due to a failure to refer for consideration of organ donation, in part, due to incorrect assumptions regarding eligibility criteria and failure of the healthcare team to refer for consideration of donation. In Ontario, over the 2017–2018 to 2018–2019 fiscal years, 33 (19%) of 178 patients who died in the ED and were not referred to the organ donation organization had organ donation potential (unpublished data, Trillium Gift of Life Network, 2019).

A potential organ donor is someone who has a very high chance of death but in whom active care continues or suitability for donation has not yet been established. In those cases where continued physiological support will have no benefit for prognostication or neurological outcome, physicians should refer patients to their provincial organ donation organization prior to WLSM. In accordance with the Potential Organ Donor Identification and System Accountability guideline,^7^ patients who meet the following criteria should be considered potential organ donors and referred to the organ donation organization:

1. Ventilated (invasive or non-invasive)
2. Condition with a grave prognosis in which death is imminent
3. Consideration of WLSM

## POSITION STATEMENT

To ensure that the management of DBI includes an observation period for optimized neuro-prognostication and that families are given the opportunity to consider organ donation as part of quality end-of-life care, the following high-level concepts are supported:

1. Early prognostication in devastating brain injury has known limitations and can be inaccurate. A sufficient period of observation and physiological support increases the opportunity for patient survival/recovery.
2. WLSM in DBI cases should be decided after observation of clinical evolution in an ICU setting in order to optimize patient outcomes. Exceptions to this would include, but are not limited to, the following case scenarios:
  ◦ It is clearly outlined that ongoing care is not consistent with the patient's previously expressed wishes (either documented or supported by available substitute decision-maker)
  ◦ Physiological futility – inability to maintain cardio-respiratory stability function due to extent of injuries or illness severity
  ◦ Concurrent comorbidities that are considered inappropriate for ICU admission in the absence of a DBI
3. Although a critical care setting with neurosurgical capacity is preferred when indicated and feasible, for patients without surgical indications, these aims could be achieved in any critical care environment.
4. Where patient survival/recovery is not possible, it provides an important opportunity to consider organ donation.
5. Identification and timely referral of potential organ donors in the ED as part of end-of-life care can save and enhance lives through organ and tissue donation.
6. Physiological support should be maintained until the following:
  ◦ The patient receives an appropriate period of observation for neuro-prognostication based on clinical circumstances
  ◦ A decision for WLSM has been made or the patient meets neurological criteria for death determination
  ◦ The patient has been referred to the organ donation organization
  ◦ A donation conversation has been facilitated, where appropriate
7. This strategy has the potential to increase the number of survivors from DBI and fulfils the opportunity to save lives through organ and tissue donation.
