# CAEP Position Statement – Management of devastating brain injuries in the emergency department: Enhancing neuroprognostication and maintaining the opportunity for organ and tissue donation – ERRATUM

**DOI:** 10.1017/cem.2020.468

**Published:** 2020-09

**Authors:** Andrew Healey, Murdoch Leeies, Carmen Hrymak, Alecs Chochinov, Brian Grunau, Bojan Paunovic, Jeanne Teitelbaum, Lindsay C. Wilson, Sam D. Shemie

The original publication of Healey et al. (2020) contained an error in the title. The article is a CAEP Position Statement, not a CAEP Joint Position Statement.

The full correct title is “CAEP Position Statement – Management of devastating brain injuries in the emergency department: Enhancing neuroprognostication and maintaining the opportunity for organ and tissue donation.”

The publisher regrets this error. The original article has been updated.
